# Determinants for HIV testing and counselling in Nairobi urban informal settlements

**DOI:** 10.1186/1471-2458-11-663

**Published:** 2011-08-23

**Authors:** Abdhalah K Ziraba, Nyovani J Madise, James K Kimani, Samuel Oti, George Mgomella, Mwau Matilu, Alex Ezeh

**Affiliations:** 1 African Population and Health Research Center, Kirawa Road, off Peponi Road P. O. Box 10787, 00100, Nairobi, Kenya; 2 Faculty of Epidemiology and Population Health, Department of Population Studies. London School of Hygiene and Tropical Medicine. Room LG21, Keppel Street, London, WC1E 7HT, UK; 3 University of Southampton, School of Social Sciences, SO17 1BJ, Southampton, UK; 4 Kenya Medical Research Institute (KEMRI). P. O. Box 54840, 00200, Nairobi, Kenya

## Abstract

**Background:**

Counselling and testing is important in HIV prevention and care. Majority of people in sub-Saharan Africa do not know their HIV status and are therefore unable to take steps to prevent infection or take up life prolonging anti-retroviral drugs in time if infected. This study aimed at exploring determinants of HIV testing and counselling in two Nairobi informal settlements.

**Methods:**

Data are derived from a cross-sectional survey nested in an ongoing demographic surveillance system. A total of 3,162 individuals responded to the interview and out of these, 82% provided a blood sample which was tested using rapid test kits. The outcome of interest in this paper was HIV testing status in the past categorised as "never tested"; "client-initiated testing and counselling (CITC)" and provider-initiated testing and counselling (PITC). Multinomial logistic regression was used to identify determinants of HIV testing.

**Results:**

Approximately 31% of all respondents had ever been tested for HIV through CITC, 22% through PITC and 42% had never been tested but indicated willingness to test. Overall, 62% of females and 38% of males had ever been tested for HIV. Males were less likely to have had CITC (OR = 0.47; p value < 0.001) and also less likely to have had PITC (OR = 0.16; p value < 0.001) compared to females. Individuals aged 20-24 years were more likely to have had either CITC or PITC compared to the other age groups. The divorced/separated/widowed were more likely (OR = 1.65; p value < 0.01) to have had CITC than their married counterparts, while the never married were less likely to have had either CITC or PITC. HIV positive individuals (OR = 1.60; p value < 0.01) and those who refused testing in the survey (OR = 1.39; p value < 0.05) were more likely to have had CITC compared to their HIV negative counterparts.

**Conclusion:**

Although the proportion of individuals ever tested in the informal settlements is similar to the national average, it remains low compared to that of Nairobi province especially among men. Key determinants of HIV testing and counselling include; gender, age, education level, HIV status and marital status. These factors need to be considered in efforts aimed at increasing participation in HIV testing.

## Background

Although the HIV/AIDS epidemic is in its third decade, many more new infections continue to occur [1]. HIV transmission in sub-Saharan Africa is predominantly heterosexual and as such a lot of prevention efforts have focused on sexual behaviour change. However, for behaviour change to happen, it is important that individuals know their HIV status so that they can take informed and appropriate action [2,3]. Voluntary Counselling and Testing (VCT) has been recommended as an effective entry point to prevention and care [4-7].

In Kenya, the official government position for HIV testing since the early days of the epidemic was premised on the client-initiated testing and counselling (CITC) model, which entails a client taking the initiative to be tested for HIV. The process involves provision of pre-testing counselling, testing and post-testing counselling with a lot of emphasis on maintaining client confidentiality. However, several years into the epidemic, the uptake of counselling and testing remained low [8-10]. For example, in an earlier survey in 2003, it was estimated that approximately 27% and 25.4% of females and males respectively, in Nairobi province, had ever been tested for HIV and received results [8]. In a later national survey in 2008, 73.8% of women and 57.3% of men in Nairobi province had ever tested and got results [10]. It is possible that there are variations in testing coverage within the slums just like it has been observed for other health indicators [11], however no study has been carried yet.

Globally, there has been an ongoing debate on the need to increase testing rates. In 2007, WHO and UNAIDS published guidelines for provider-initiated testing and counselling (PITC), to compliment the traditional client-initiated testing and counselling [12]. With increasing availability of relatively effective interventions such as prevention of mother-to-child transmission (PMTCT) and anti-retroviral therapy (ART), as incentives, provider-initiated testing and counselling is being promoted as a way of increasing testing coverage in several countries, including Kenya which published the first guidelines in 2008 for this purpose [13]. In the PITC model, individuals presenting to a health facility are encouraged to be tested for HIV as part of the routine medical investigation, but are free to opt-out of the testing if they so wish.

While Kenya is among the countries with relatively high coverage of HIV testing (37%) in sub-Saharan Africa, this is still low [14]. One of the most likely explanations for the poor uptake of HIV/AIDS testing services in Kenya and many developing countries is related to the high levels of stigma associated with HIV/AIDS[15-17]. Consequently, many people continue to die without knowing their status and without accessing HIV/AIDS treatment, HIV positive mothers continue to give birth without using PMTCT services [18] and many discordant couples are not aware of partner's HIV status to enable them to take action [19-21]. Those who seek testing and counselling tend to do it late when they are symptomatic or perceive themselves to be at a high risk of HIV infection [22-26]. Socio-demographic characteristics such as gender (i.e. females), young age, higher education, and higher socioeconomic status have been reported to be associated with use of testing and counselling, but no study has been carried in the informal settlements [27-29].

Clinic-based studies on testing behaviour, which are the commonest, fail to capture views and characteristics of those who do not go for testing. Secondly, prior studies have not discerned predictors for CITC from those of PITC and yet these are likely to be different. This study uses data from testers and non-testers to examine prior HIV testing status, identify unmet need for testing, and predictors of HIV testing. We hope that our results will help inform HIV prevention and care programs to adjust their activities to reach people who do not know their HIV status and hopefully reduce risk of new infections, and promote early initiation of treatment among those who are infected.

## Methods

We used data from a cross-sectional population-based sero-survey nested in a longitudinal framework (Nairobi Urban Health and Demographic Surveillance System-NUHDSS) run by the African Population and Health Research Center (APHRC). The sero-survey was implemented in collaboration with the Kenya Medical Research Institute (KEMRI). The survey was conducted in two slums; Viwandani and Korogocho between 2006 and 2007.

A simple random sample of 5,004 respondents estimated to be adequate to answer the key research questions was drawn from the NUHDSS database of over 60,000 individuals for testing. After updating the residence status in a subsequent NUHDSS data collection round, 237 of those sampled were confirmed to have been non-residents at the time of sampling and were dropped from the final sample. Thus the effective sample was 4,767 (5,004 minus 237). Out of the sample of 4,767, 3,162 individuals (66.3%) consented to be interviewed, 2,721 (57.1%) provided a blood sample while 2,590 (54.3%) provided both interview and a blood sample. Participants had the option of accepting the interview only, the blood test only, accepting both or refusing both. Participation in the research was limited to females aged 15-49 years and males 15-54 years. We took a higher upper age limit for males because HIV prevalence among males tend to peak at a later age compared to women. Trained counsellors and phlebotomists interviewed respondents and drew samples respectively. The study received ethical approval from the Kenya Medical Research Institute's Scientific and Ethical Review committees. Proper consent procedures were observed for all participants prior to participation.

### Data collection and Measurements

A list of all sampled clients was generated with enough information to enable field workers to positively identify the respondents. On the other hand, the questionnaire and blood sample filter papers did not contain any identifiers except a new ID that enabled linkage to the NUHDSS data. Interviews were conducted using a pre-tested and structured questionnaire. For individuals who had had more than one testing event in the past, data presented here only refers to the latest test event.

Blood sample collection involved aseptically collecting drops of blood from a finger prick using diabetic pen-devices with non-reusable lancets. For each participant, 5 drops of blood were collected and air dried on a filter-paper card. Each dried blood spot (DBS) sample was labelled with a unique identification number for enabling creation of a link between the sero-data, survey data and the individual demographic surveillance data from the NUHDSS database. Participants who wanted to know their HIV status were referred to our collaborating VCT providers for counselling and testing services. HIV serology on DBS was carried out using Determine^® ^HIV-1/HIV-2 (Abbott) and Uni-Gold™ rapid test kits.

### Data analysis

Descriptive and multivariate analyses were carried out using Stata statistical software version 10. The dependent variable was categorised as trichotomous thus: Never tested; Client-Initiated Testing and Counselling (CITC) and Provider-Initiated Testing and Counselling-(PITC). Tests carried out for purposes of emigration, employment or travel were very few (3.7%) and for analytical purposes these were grouped together with provider-initiated testing and counselling. Individuals who had never tested for HIV were further asked whether they would like to be tested. Responses from those who indicated that they want to be tested were used to measure the unmet need for testing and counselling. In this regard, unmet need for HIV testing in this paper is defined as the proportion of respondents who have never had testing and counselling for HIV, but would like to be tested.

Multinomial logistic regression models were fitted to identify determinants of HIV testing status. We ran several bivariate models and identified variables that were significantly associated with the outcome at 10% level of significance and included them in the multinomial regression analysis. In addition, we also included variables that have been reported in the literature to be associated with the outcome variable even if they were not significant at bivariate analysis. Variables that were used in the final model included; stigma, mobility, HIV/AIDS-related knowledge, HIV status, opinion on testing, knowledge of a facility that offers HIV/AIDS-related services, risky sexual behaviour, age at sexual debut, education level, ethnicity, marital status, wealth, slum of residence, age and sex. The estimates in the final model for each of the variables are adjusted, controlling for all the other variables.

Stigma was computed as a score from a set of four questions, three on avoidance and one on secrecy about HIV thus: *Would you buy fresh vegetables from a vendor who has the virus that causes AIDS? If a female teacher has the virus that causes AIDS, should she be allowed to continue teaching in school? If a relative of yours became sick with the virus that causes AIDS, would you be willing to care for her or him in your own home? If a member of your family got infected with the virus that causes AIDS, would you want it to remain a secret? *After taking careful consideration of the direction of the questions and the responses given, we found that Cronbach's alpha was low -less than 50%. Also, because of the limited dimensionality of the data (4 questions) we felt that use of principal component analysis (PCA) would not be a good idea. As a last resort, we measured stigma as a count score based on the four questions asked.

The mobility index was taken to be a weighted count of movement episodes of individuals within or outside the demographic surveillance area per unit time. An individual was considered to be highly mobile if they had at least one or more episodes of internal change of residence per year or at least one outmigration and return episode to the surveillance area in two years. HIV/AIDS-related knowledge index was computed from a set of questions about knowledge of HIV transmission; prevention and misconceptions similar to those used in Demographic and Health Surveys (DHS) [8] using PCA. Household wealth index was computed using PCA and the items used for the computation included ownership of television, radio, bicycle and amenities such as water, toilet, and house building materials. Risky sexual behaviour was measured using one question on multiple sexual partnerships. An individual was classified as having had high risk sexual activity if they had two or more sexual partners in the last twelve months before the survey. Respondent's opinion about HIV testing was based on a single question *"Do you think it is important for people to know if they have the virus that causes AIDS"*.

We carried out a Wald's test using a user written program implemented in Stata (*spost) *using the command *mlogtest *to determine whether any of the three categories of the outcome variable could be combined with another. The null hypothesis being tested was "all coefficients except intercepts associated with a given pair of outcomes are zero (i.e. categories can be collapsed)" [30]. Each pair of outcomes was significantly different: Never tested Vs CITC-chi square = 136, df = 35, p value < 0.001; Never tested Vs PITC-chi square = 239, df = 36, p value < 0.001; and CITC Vs PITC-chi square = 113, df = 36, p value < 0.001, indicating that the categories could not possibly be collapsed hence the appropriateness of multinomial logistic model.

## Results

Approximately 31% of participants who were interviewed had ever been tested for HIV in the past through CITC, 22% through PITC while the rest had never been tested (see Table 1). The highest proportions of individuals who had CITC were recorded among the age categories of 20-24, 25-29 and 30-34 years. More females (33%) had ever been tested through CITC compared to 29% among males. The divorced/separated/widowed also had higher proportions of individuals who had had CITC (43%) compared to 31% among those who were currently married. Approximately 57% of individuals with higher education had been tested through CITC compared to only 29% among those with no formal education. Among all identifiable groups, individuals between the age of 20 to 35 years, females, the married and those with no formal education tended to have been tested through PITC compared to others groups. Overall, CITC was higher in almost all identifiable groups than PITC with exceptions of "other ethnicity" and "unknown educational level", (see Table 1).

**Table 1:** Proportion of individuals ever tested for HIV by socio-demographic characteristics

Variables	Total Number	Never tested	CITC	PITC	Total	*X*^2 ^(p value)
Age group						
<20 yrs	443	63.7	21.0	15.4	100.0	124.2 (<0.001)
20-24 yrs	732	38.5	35.3	26.2	100.0	
25-29 yrs	662	38.1	36.1	25.8	100.0	
30-34 yrs	493	43.2	34.3	22.5	100.0	
35-39 yrs	383	52.2	29.2	18.5	100.0	
40-44 yrs	244	56.2	25.0	18.9	100.0	
45-49 yrs	161	60.9	24.8	14.3	100.0	
50+ yrs	44	56.8	31.8	11.4	100.0	
Gender						
Female	1,998	38.2	32.5	29.2	100.0	234.1(<0.001)
Male	1,164	62.3	28.9	8.9	100.0	
Marital status						
Married	2,062	43.0	30.7	26.3	100.0	140.1 (<0.001)
Divorced/separated/widowed	323	36.5	43.0	20.4	100.0	
Never married	777	62.3	27.7	10.0	100.0	
Residence						
Korogocho	1,756	48.7	30.8	20.6	100.0	4.9 (0.088)
Viwandani	1,406	45.1	31.7	23.2	100.0	
Ethnicity						
Kikuyu	981	44.6	34.4	21.1	100.0	28.9 (<0.001)
Luhya	493	52.7	28.2	19.1	100.0	
Luo	567	48.3	30.9	20.8	100.0	
Kamba	663	46.8	32.9	20.4	100.0	
Others	458	45.4	25.6	29.0	100.0	
Education						
No schooling	119	43.7	28.6	27.7	100.0	18.9 (0.015)
Primary	2,015	48.7	29.7	21.6	100.0	
Secondary	952	44.9	34.4	20.8	100.0	
Higher	23	21.7	56.5	21.7	100.0	
Don't know	53	45.3	26.4	28.3	100.0	
Wealth Index						
Poorest 20%	441	49.9	32.7	17.5	100.0	15.6 (0.048)
2nd	611	47.6	32.9	19.5	100.0	
3rd	684	49.7	29.0	21.4	100.0	
4th	683	43.5	31.3	25.2	100.0	
Richest 20%	743	45.9	30.8	23.3	100.0	
Mobility Index						
Not highly mobile	2820	47.9	30.7	21.4	100.0	7.6 (0.022)
Highly mobile	342	40.1	35.1	24.9	100.0	
Total	3,162	47.1	31.2	21.7	100.0	

Looking at overall coverage of testing and counselling i.e. sum of CITC and PITC, the highest percentages of individuals who had ever been tested were those in the age bracket 20 to 34 years (Table 1). More women (62%) had ever been tested compared to men (38%). By marital status, the divorced/separated/widowed had a higher proportion of individuals who had ever been tested (64%) compared to those who had never married (38%). The proportion of individuals who had ever had HIV testing and counselling increased with educational attainment from 51% among those with primary education to 55% among those with secondary education and 78% among those with higher education. However the proportion of individuals ever tested among those with no formal education was higher (56%) than that of individuals with primary or secondary education. There were no significant differences in proportions of individuals who had ever been tested by wealth status index. A higher proportion of individuals (60%) categorised as highly mobile had had testing and counselling before than the less mobile ones (52%).

**Figure 1:**
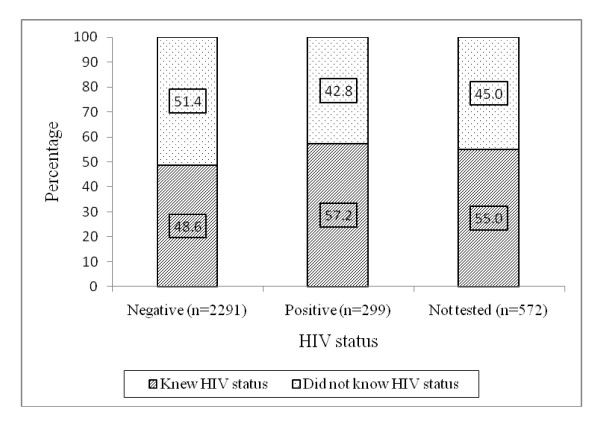
**Percentage distribution of prior knowledge of one's HIV status by current HIV status as established in this study**.

Figure 1 shows the proportion of those with prior knowledge of their HIV status and the current HIV status as determined in this study. Among individuals who tested negative, 51.4% did not know their status at the time of the study. Among those who tested positive, approximately 43% did not know their HIV status while among those who did not test in this study, 45% did not have knowledge of their HIV status.

Table 2 shows a further breakdown of those who had never been tested by intention to test in the future. Overall, approximately 5% of respondents did not intend to test at all in the future (Table 2). The proportion of individuals who did not intend to test seems to increase with age and was higher among males, residents of Korogocho and among those with no formal education. The unmet need for HIV testing (proportion of all respondents who had never been tested and desired to be tested) was about 42% and was highest among younger individuals under 20 and over 40 years of age, among those who have never been married, males and was least among those with tertiary level education.

**Table 2:** Percentage distribution of HIV testing status: Future testing intentions, unmet testing need, CITC and PITC by socio-demographic characteristics

Variables	Total Number	Never tested		Ever tested	
			
		Does not **want test**, N = 153	Wants to **test**, N = 1,336	**CITC**, N = 986	**PITC**, N = 687
Age group					
<20 yrs	443	3.6	60.1	21.0	15.4
20-24 yrs	732	4.0	34.6	35.3	26.2
25-29 yrs	662	3.9	34.1	36.1	25.8
30-34 yrs	493	4.7	38.5	34.3	22.5
35-39 yrs	383	7.6	44.7	29.2	18.5
40-44 yrs	244	7.8	48.4	25.0	18.9
45-49 yrs	161	5.0	55.9	24.8	14.3
50+ yrs	44	6.8	50.0	31.8	11.4
Gender					
Female	1,998	3.9	34.4	32.5	29.2
Male	1,164	6.5	55.8	28.9	8.9
Marital status					
Married	2,062	5.2	37.8	30.7	26.3
Divorced/separated/widowed	323	4.3	32.2	43.0	20.4
Never married	777	4.0	58.3	27.7	10.0
Residence					
Korogocho	1,756	5.6	43.1	30.8	20.6
Viwandani	1,406	3.9	41.2	31.7	23.2
Ethnicity					
Kikuyu	981	5.4	39.1	34.4	21.1
Luhya	493	5.9	46.9	28.2	19.1
Luo	567	4.4	43.9	30.9	20.8
Kamba	663	3.0	43.7	32.9	20.4
Others	458	5.7	39.7	25.6	29.0
Education					
No schooling	119	8.4	35.3	28.6	27.7
Primary	2,015	4.6	44.1	29.7	21.6
Secondary	952	4.6	40.2	34.4	20.8
Higher	23	4.4	17.4	56.5	21.7
Don't know	53	9.4	35.9	26.4	28.3
Wealth Index					
Poorest 20%	441	4.5	45.4	32.7	17.5
2nd	611	6.4	41.2	32.9	19.5
3rd	684	4.0	45.8	29.0	21.4
4th	683	4.8	38.7	31.3	25.2
Richest 20%	743	4.6	41.3	30.8	23.3
Mobility Index					
Not highly mobile	2820	5.11	42.84	30.71	21.35
Highly mobile	342	2.63	37.43	35.09	24.85
Total	3,162	4.8	42.3	31.2	21.7

Table 3 shows multivariate analysis results for determinants of HIV testing status with "never tested" as the base outcome- that is, PITC and CITC are being compared with those who had never tested. The estimates presented here for each variable are adjusted, controlling for all other variables in the model. Males had significantly lower odds of having had CITC (OR 0.47; p value < 0.001) and PITC (OR 0.16; p value < 0.001) compared to females holding all else constant. Generally, controlling for all other variables, the odds of testing tended to decrease with age for both CITC and PITC although non-significant results were observed for age categories 20-29 years and 50 or more years. Individuals with secondary and higher education had higher odds of having had CITC compared to their counterparts with primary education level but there were no significant differences for PITC. The Luhya ethnic group compared to the Kikuyu ethnic group were significantly less likely to have previously been tested for HIV through either CITC (OR 0.65; 95% CI, 0.48-0.87) or PITC (OR 0.67; 95% CI, 0.47-0.95). Divorced/widowed or separated individuals had approximately 65% higher odds of having CITC than married individuals. The never married had lower odds of having had CITC (OR 0.71; 95% CI, 0.54-0.93) and PITC (OR 0.28; 95% CI, 0.19-0.40) compared to the married.

**Table 3:** Multinomial logistic regression model for determinants of "HIV testing status"

	Not tested Vs CITC	Not tested Vs PITC
		
Variables	OR [95% CI]	OR [95% CI]
Gender (Ref = Female)		
Male	0.47*** [0.38,0.58]	0.16*** [0.12,0.21]
Slum (Ref = Korogocho)		
Viwandani	0.94 [0.75,1.17]	1.07 [0.83,1.39]
Age category (Ref = 20-24 yrs)		
<20 yrs	0.59** [0.40,0.87]	0.76 [0.48,1.20]
25-29 yrs	0.83 [0.62,1.12]	0.84 [0.61,1.16]
30-34 yrs	0.71* [0.52,0.98]	0.65* [0.45,0.94]
35-39 yrs	0.44*** [0.31,0.62]	0.43*** [0.29,0.64]
40-44 yrs	0.35*** [0.23,0.54]	0.41*** [0.26,0.66]
45-49 yrs	0.34*** [0.21,0.55]	0.32*** [0.18,0.59]
50+ yrs	0.58 [0.27,1.26]	0.77 [0.27,2.18]
Education (Ref = Primary)		
No schooling	1.50 [0.85,2.68]	1.47 [0.79,2.73]
Secondary	1.26* [1.01,1.57]	1.09 [0.84,1.42]
Higher	3.04* [1.00,9.18]	2.19 [0.53,8.97]
Don't know	0.82 [0.36,1.84]	1.36 [0.61,3.03]
Ethnicity (Ref = Kikuyu)		
Luhya	0.65** [0.48,0.87]	0.67* [0.47,0.95]
Luo	0.79 [0.59,1.05]	0.88 [0.63,1.24]
Kamba	0.96 [0.72,1.27]	0.89 [0.64,1.25]
Others	0.83 [0.59,1.16]	1.31 [0.90,1.89]
Marital Status (Ref = Married)		
Divorced/separated/widowed	1.65** [1.19,2.27]	0.94 [0.64,1.37]
Never married	0.71* [0.54,0.93]	0.28*** [0.19,0.40]
Wealth Index (Ref = Richest)		
Poorest 20%	1.09 [0.78,1.51]	0.92 [0.61,1.37]
2nd	1.17 [0.87,1.59]	1.16 [0.82,1.64]
3rd	0.97 [0.73,1.30]	0.94 [0.68,1.32]
4th	1.18 [0.88,1.57]	1.25 [0.90,1.72]
Mobility (Ref = Less mobile)		
Highly mobile	1.13 [0.83,1.54]	1.02 [0.72,1.45]
Stigma Index (Ref = Least stigmatising)		
Most stigmatising	0.65** [0.49,0.86]	0.76 [0.55,1.04]
Moderate	0.88 [0.71,1.09]	0.84 [0.65,1.07]
HIV status (Ref = Negative)		
Positive	1.60** [1.15,2.22]	1.30 [0.89,1.92]
Not known	1.39* [1.08,1.80]	1.29 [0.96,1.73]
HIV Knowledge level (Ref = 2nd)		
Lowest	0.64** [0.48,0.84]	0.79 [0.58,1.08]
3rd	1.04 [0.77,1.42]	0.81 [0.55,1.18]
4th	0.90 [0.67,1.22]	0.92 [0.65,1.30]
Highest	0.86 [0.66,1.12]	0.75 [0.54,1.03]
Good to know HIV status (Ref = Yes)		
No	0.32*** [0.16,0.63]	0.16*** [0.06,0.43]
Knows HIV/AIDS facility (Ref = Yes)		
No	0.60*** [0.48,0.74]	0.79* [0.62,1.00]
Sex with other partners in 1 year (Ref = No)		
Yes	0.87 [0.60,1.27]	0.79 [0.45,1.38]
Age at sex debut (Ref = Less than 15 yrs)		
15-19 yrs	0.95 [0.73,1.24]	1.18 [0.84,1.65]
20 + yrs	1.02 [0.73,1.42]	1.62* [1.07,2.44]

* p < 0.05; ** p < 0.01; *** p < 0.001

Individuals who were found to be HIV-positive in this study and those whose HIV status was not determined had higher odds of having had CITC (OR 1.60; 95% CI, 1.15-2.22 and OR 1.39; 95% CI, 1.08-1.80), respectively, compared to those who were found to be HIV-negative. Individuals ranked as being among the lowest 20% on HIV/AIDS-related knowledge index had lower odds of having had CITC compared to those in the second 20% (OR 0.64, p value < 0.01). The differences were not significant for rest of the knowledge index categories. Individuals who thought that knowing one's HIV status was not good were less likely to have had either CITC (OR 0.32; 95% CI, 0.16-0.63) or PITC (OR 0.16; 95% CI, 0.06-0.43) compared to those who thought it was good to know one's HIV status. Lack of knowledge of location of health care facilities that offer HIV/AIDS-related services was significantly associated with lower odds of both CITC (OR 0.60; 95% CI, 0.48-0.74) and PITC (OR 0.79; 95% CI, 0.62-0.99). Wealth status index, area of residence and multiple sexual partnerships were neither associated with CITC nor PITC.

## Discussion

The focus of this paper was to examine the state of HIV testing and counselling and its determinants in Nairobi informal settlements. It is widely acknowledged that knowing one's HIV status is a major step in the prevention, initiation of treatment with antiretroviral drugs, prevention and treatment of opportunistic infections and use of psychosocial support services. In spite of this, many individuals in sub-Saharan do not know their HIV status. Our results corroborated those of previous studies which found uptake of voluntary counselling and testing services to be low [8,9,31]. Compared to Nairobi city as a whole, slum settlements have a lower proportion of women and men who have ever been tested for HIV. This underscores the challenges that are faced by slum residents with regard to the access and utilization of health care services. In the sub-Saharan region, Kenya has one of the highest proportions of individuals who have ever tested for HIV with about 70% of urban women and 52% of rural women having ever tested. The corresponding figure for Uganda stands at 40.7% for urban women and 21.6% for rural women, while for Tanzania 24.7% of urban women and only 7.1% of rural women have ever tested [10,32,33]. Our study showed that a substantial proportion of those who have never tested would like to do so and this gives hope that eventually most people will get tested if efforts of reaching them are intensified.

As often happens with other forms of health care utilization, there were significant differences in testing and counselling by socio-demographic characteristics. For example, gender differentials were confirmed in the multivariable analyses where the findings showed that women were more likely to have had either type of testing and counselling compared to men. An earlier national survey carried out in 2003 showed that only 13.1% of women and 14.3% of men in Kenya had been tested at the time of the survey, while the most recent national survey showed that 56.5% of women and 39.9% of men had ever been tested and received results [10]. The apparently widening gap for HIV testing and counselling between women and men might be due to increased testing among women in PMTCT programs. It is also not clear whether the very low levels of PITC among men is majorly an issue of women having more contact with the health care system or men are more likely to opt out of PITC when it is offered.

Secondary or higher education was found to be associated with higher likelihood of having had CITC. This is in line with a common observation that individuals with higher education levels tend to use more health care services as compared to those with low or no formal education [27,34,35]. This observation did not however hold true for PITC and it is not obviously clear why this was the case. As expected, low HIV/AIDS-related knowledge was associated with lower usage of CITC- but not PITC, since knowledge is important in initiating and making a decision to seek care. These findings suggest that when a client is offered the option of getting tested, their chances of accepting to participate are neither influenced by their educational level attainment nor prior HIV/AIDS-related knowledge levels.

Marital status was also an important determinant of HIV testing. Not only can an HIV-positive result lead to divorce or separation [36], the death of a spouse from suspected AIDS may motivate the surviving spouse to seek testing. This might explain the finding observed in this study that divorced/separated/widowed individuals were more likely to have had CITC compared to married individuals. The finding that CITC among married individuals was low might be related to the false notion that people in stable marital partnerships are at a lower risk of contracting HIV yet recent evidence suggest that most new infections are taking place among married individuals who were previously thought to be a low risk group [37,38]. The significantly lower likelihood of the never married women having had PITC might be a reflection of their limited contact with the health care system as compared to their married counterparts who mainly make contact for obstetric reasons.

The finding that those who were HIV-positive were more likely to have been tested before confirms earlier evidence that individuals who feel that they are at risk, have been exposed or are symptomatic are likely to seek testing [26]. This is a trend prevention programs would not want to see continue in a generalised epidemic because individuals who perceive themselves to be at low risk or asymptomatic are not necessarily uninfected and might continue to fan the epidemic.

The Luhya ethnic group was less likely to have had either CITC or PITC compared to Kikuyu and yet in this population the Luhya have the second highest HIV prevalence at 12% compared to 8% among the Kikuyu ethnic group in the slums[39]. We could not find a plausible explanation, so future ethnographic research should investigate this observation. While wealth has been found to be associated with use of testing and counselling in other studies, this was not the case in this population [40,41]. This might be related to the fact that there is little variability in terms of wealth among the urban poor residents in the slums, but also the fact that the monetary cost involved in getting counselling and testing is too minimal to be a barrier to accessing the service.

Overall, there are two main policy implications of our findings: i) the need to increase overall testing coverage and ii) the need to address disparities in HIV testing across social groups. While there has been some improvement in the proportion of individuals ever tested over the years, a substantial proportion have never tested and do not know their status and thus need to be reached. With approximately 41% of all those ever tested having been tested through PITC, proponents of integration of HIV testing into routine medical care would welcome this observation and recommend its promotion further. Also, based on our results, it appears that men are being left behind in terms of HIV testing. Given the high level of sero-discordance in Kenya as reported in an earlier survey and the low level of awareness of its existence, targeting couples for testing and counselling might help increase proportion of males who get tested and thus potentially reduce the risk of new infections [9]. A strategy, such as encouraging male partners to accompany their partners and participate in PMTCT antenatal clinics could help increase the proportion of men testing and strengthen their role as partners in the fight against the epidemic.

## Limitations

In this study, information about HIV-testing status was based on self reported responses. This could be potentially affected by recall bias if the test was offered a long time ago, and it could also be affected by the tendency of respondents preferring to give socially desirable answers. Measurement of stigma, a key variable, may not have been satisfactory due to insufficient questions to cover the various dimensions of HIV/AIDS-related stigma. The HIV-testing status was taken with no time boundaries (ever tested) and yet individual's HIV status can change in a matter of weeks. At the time of designing the study, it was envisaged that few people had ever tested and even fewer would have been tested in the last one year. Besides, from a program point of view, it can be argued that knowledge of a test result is likely to have a relatively long-term impact on behaviour probably stretching beyond the commonly arbitrarily set cut-off of one year. Lastly, there was potential selection bias given that a substantial proportion of the sample initially targeted did not participate in the study. However an assessment of potential bias in the HIV prevalence estimates due to non-participation showed that selection bias was unlikely because overall HIV prevalence was overestimated by only 2% (overestimated by 3% among women and underestimated by 1% among males) [39].

## Conclusions

The proportion of individuals who have ever had testing and counselling in the two informal settlements is lower-(62% for women and 38% for men) than that of Nairobi as a whole (76% for women and 60% for men), but more comparable to the national average (58.4% for women and 42% for men) over the period 2003-2008 [10]. The unmet need for testing and counselling is high and, therefore, programs should intensify their efforts to reach out to this sub-population. The main determinants for HIV counselling and testing were; gender, age, marital status, ethnicity, and favourable attitude towards knowing one's status. Other less strongly associated factors include HIV related knowledge and HIV/AIDS-related stigma. Our results further showed that the determinants for PITC and CITC are not exactly the same. For example, while secondary or higher education; and positive or unknown status were associated with higher tendency to use CITC, these factors did not matter for PITC. Also, while individuals with the most stigmatising tendency were less likely to have had CITC, levels of one's stigma did not influence use of PITC. These differences may need to put into consideration while trying to promote up take of testing and counselling. Provider-initiated testing and counselling constitutes a sizeable proportion of all those tested and seems to be helping to increase overall testing rates and should be promoted more in the future.

## Conflict of interests

The authors declare that they have no competing interests.

## Authors' contributions

AKZ took lead in preparing the manuscript. He participated in designing, implementing, data management and analysis and manuscript writing. NM participated in proposal development, study design, project implementation, supervision and manuscript preparation. JKK participated in data analysis and manuscript preparation. MM participated in the proposal development, study design, project implementation, supervision of blood sample testing and manuscript preparation. SO participated in writing of the background section, interpretation of results and writing of the discussion section. GM participated in writing the methods and discussion sections and provided overall support for the manuscript preparation. AE participated in the proposal development, study design, project implementation, supervision and overall project management. He also participated in manuscript preparation. All authors read and approved the final manuscript.

## Pre-publication history

The pre-publication history for this paper can be accessed here:

http://www.biomedcentral.com/1471-2458/11/663/prepub
